# Supplementary material for: Transthyretin Is Commonly Upregulated in the Hippocampus of Two Stress-Induced Depression Mouse Models
Source: Int J Mol Sci. 2023 Feb 13;24(4):3736. doi: 10.3390/ijms24043736 (PMC9964880; doi:10.3390/ijms24043736)
Supplement: Supplementary file 1 [file ijms-24-03736-s001.zip › ijms-2154513-supplementary.pdf]

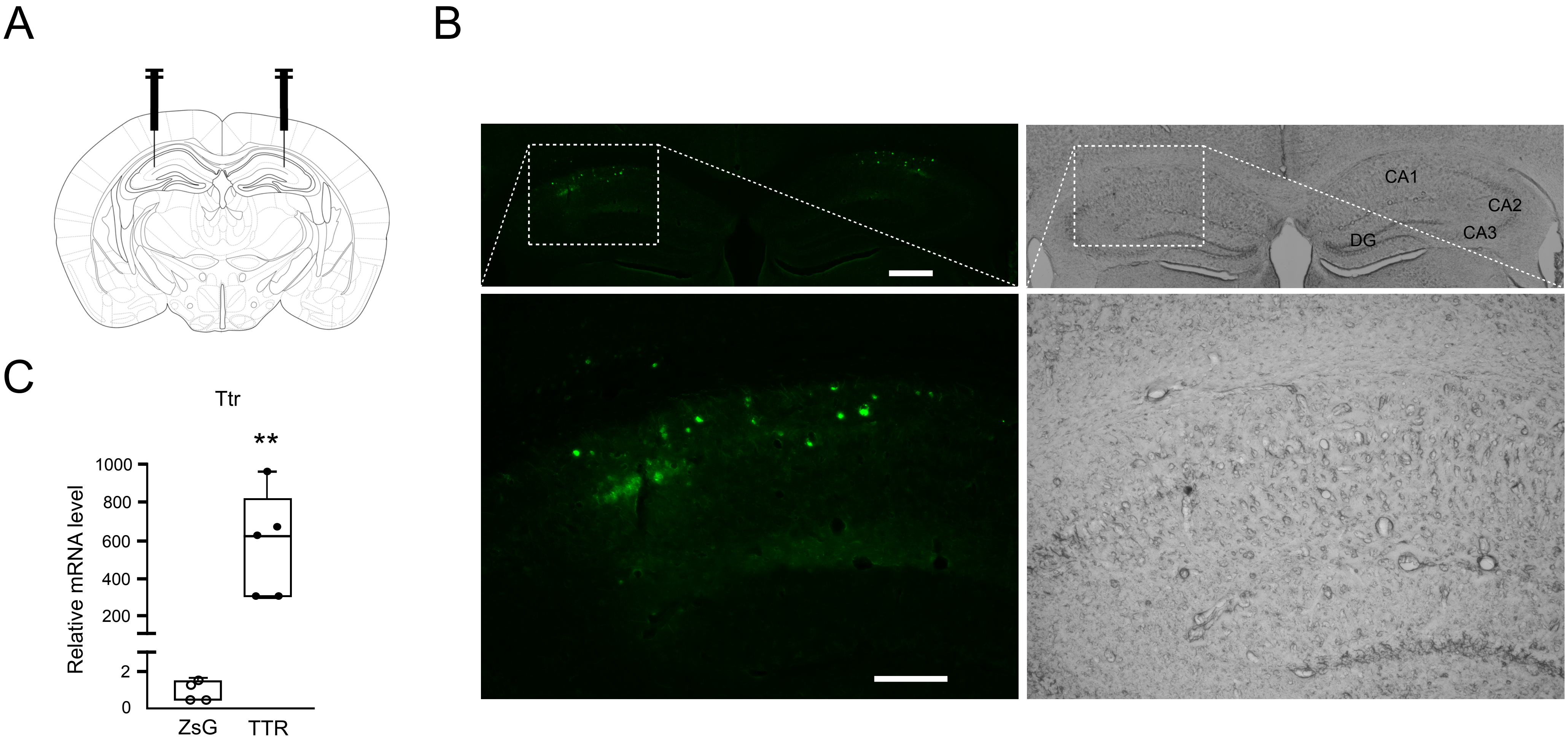

Supplementary Figure S1. Image and mRNA level of gene overexpression in the hippocampus. (A) Schematic showing placement of AAV-ZsGreen1 and AAV-Ttr injection sites in coronal slice of the mouse brain. (B) Representative image of ZsGreen1 overexpression with AAV-ZsGreen1 in the hippocampus of male B6 mice with high magnification shown as lower layer. Scale bar: 500 μM (upper layer), 200 μM (lower layer). DG, dentate gyrus. (C) The mRNA level of Ttr was analyzed by RT-qPCR with RNA extracted from the hippocampus of ZsG and TTR mice (n = 4–5). The box represents the 25–75th percentiles, and the median is indicated. The whiskers show the maximum and minimum values. **p < 0.01.
